# Community-Based Seroprevelance of SARS-CoV-2 in Saudi Arabia

**DOI:** 10.7759/cureus.32419

**Published:** 2022-12-11

**Authors:** Lujain Alassaf, Sami Almudarra, Abdullah Almudaiheem, Hind Almutlaq, Ada Alqunaibet, Haleemah Alseraihi, Rehab Alaswad, Abdullah T Khoja, Khaled AlAbdulkareem, Faisal AlSaif, Raghib Abu Saris

**Affiliations:** 1 Population Health Management, Center for National Health Insurance, Riyadh, SAU; 2 Gulf Center for Disease Prevention and Control, Gulf Health Council, Riyadh, SAU; 3 Public Health Intelligence, Public Health Authority, Riyadh, SAU; 4 Public Health intelligence, Public Health Authority, Riyadh, SAU; 5 Infectious Disease Consultant, Ministry of Health, Riyadh, SAU; 6 Public Health and Preventive Medicine, Imam Mohammad Ibn Saud Islamic University, Riyadh, SAU; 7 Primary Health Care, Ministry of Health, Riyadh, SAU; 8 Department of Surgery. College of Medicine, King Saud University, Riyadh, SAU; 9 Department of Epidemiology and Biostatistics, College of Public Health and Health Informatics, King Saud Bin Abdulaziz University for Health Sciences, Riyadh, SAU

**Keywords:** saudi arabia, serological survey, seroprevalence, covid-19, sars-cov-2

## Abstract

Introduction

The new coronavirus disease 2019 (COVID-19) is a major global concern. Due to the number of asymptomatic cases that go untested, the actual proportion of those who have been infected is likely to be higher than the reported prevalence. Thus, investigating the exact proportion of those who developed antibodies against the virus through serological surveys is crucial to identify the immune status of the population and direct public health decisions accordingly.

Objectives

The aim of this study is to estimate the seroprevalence of SARS-CoV-2 in the community and to describe the epidemiological characteristics of the discovered cases.

Methods

Between July and October 2020, a cross-sectional sero-survey was conducted including a total of 15,873 serum samples collected from seven regions within the kingdom. Using a multistage convenient sampling, people were invited to participate in an interviewer-administrated questionnaire. Afterward, blood samples were collected and seroprevalence was determined using the SARS-CoV-2 virus IgG/IgM antibody detection kits (ELISA). A p-value of <0.05 and 95% CI were used to report the significance.

Results

The overall seroprevalence of SARS-CoV-2 in the sample was 17.0%, and Makkah region constituted the highest number of reactive cases (33.3%). There was a significant association between all comorbidities and having symptoms except for diabetes. In addition, age, education, nationality, and region were all significant predeterminants of sero-result. Also, contact with a confirmed or suspected case increased the risk of being seropositive by nearly 1.5 times.

Conclusion

This study estimated the national seroprevalence of SARS-CoV-2 in Saudi Arabia to be 17%. At the time of this study, most of the population did not have the SARS-CoV-2 specific antibodies. This suggests that the population is still below the threshold of herd immunity and emphasizes the importance of mass vaccination programs and abiding by recommended prevention precautions.

## Introduction

As of early 2020, a new coronavirus disease caused by the novel severe acute respiratory syndrome coronavirus 2 (SARS-CoV-2) has overwhelmed the world with millions of cases to date [1]. The virus emerged first in December 2019 exactly in Wuhan, China, and later on spread under the official name “COVID-19” [2]. The novel virus belongs to the same family as the previously identified coronavirus in 2002 (SARS-CoV-1) and is believed to share some similarities with regard to pathogenesis [3]. The main route of transmission is respiratory droplets, which can be directly transmitted from person to person through talking, sneezing, or coughing. In addition, touching infected surfaces and then touching the face can be another possible, although less likely, route of transmission [4]. The testing of the causative COVID-19 virus is mainly conducted through real-time polymerase chain reaction (RT-PCR) method, which is considered the gold standard for the detection of several viruses due to its fast diagnosis, high sensitivity, and specificity [5]. More testing procedures that are less commonly used are viral testing for current infection and antibodies (serology) testing for prior infection [6]. The virus can infect people of all ages with different severity levels, and even though research is still going to provide a further understanding of the determinants and factors that influence its prognosis, early studies suggest that older age, being male, existing chronic illnesses, and smoking status are significant predictors of the severity of the disease and admission to the hospital [7,8]. Infected individuals report a variety of symptoms, with the most common being fever, dry cough, and fatigue. Other symptoms such as loss of taste or smell, rash, diarrhea, and conjunctivitis are less common [9]. However, several studies suggest that the majority of the cases have mild to no symptoms at all, yet it can spread the infection just as efficiently [10]. Thus, chasing individuals who have symptoms and are in contact with confirmed symptomatic cases might not be accurate in reflecting the overall proportion of the infected population as many mild and asymptomatic cases never get tested [11]. Hence, a more comprehensive approach to reflect the prevalence of COVID-19 infection in the community is through the detection of SARS-CoV-2 specific antibodies in their blood through serology testing, known as seroprevalence [12].

A study was conducted in the epicenter of the pandemic in China to assess the seroprevalence of COVID-19 among different sub-cohorts. Surprisingly, it was found that only 3.2% to 3.8% of the population in Wuhan tested positive for the presence of immunoglobulin G (IgG) and immunoglobulin M (IgM). The seropositivity was significantly higher for those aged 65 and above, and no statistical difference was found between males and females. Also, healthcare workers and patients who had visited hospitals significantly increased their risk of being seropositive by 3.3% compared to those who did not [13]. In Stockholm, Sweden, the seroprevalence of SARS-CoV-2 antibodies was reported to be 7.3%. Contrary to the Chinese study, higher seropositivity was observed among children and adults compared to the elderly (4.7%, 6.7%, and 2.73%, respectively). The high seropositivity rate in children and adults could be due to the fact that Sweden was not on lockdown at the time of this study as schools were kept open and remote working was not yet reinforced [14]. In another study, 1,702 participants from Los Angeles were enrolled and tested for the presence of antibodies against COVID-19. In the recruited sample, the unweighted and weighted seroprevalence of SARS-CoV-2 was 4.34% and 4.65%%, respectively, after adjusting for test diagnostics. However, the estimates of this study could be biased as the symptomatic patients were more likely to participate [15]. In Switzerland, a population-based immuno-survey was established to estimate the seroprevalence at different time points. Around 1,300 individuals were enrolled each week and were only eligible to participate once. In the first week, the seroprevalence was estimated to be 4.8%, which then increased notably in the second and third weeks to 8.5%-10.9%. The proportion of those who tested positive declined around the fourth week, indicating 6.6% seroprevalence, which then increased again in the last measurement, accounting for an overall 10.8% seroprevalence during the fifth week. Furthermore, young children (5-9) years and elderly (≥65 years) had a significantly lower seropositivity rate when compared to those aged 20-49 years (p<0.05). No significant differences were observed between males and females [16]. In Korea, the seroprevalence was reported to be only 0.07%. This remarkably low seroprevalence could be attributed to the country’s quick and early response to compact the spread of COVID-19 [17]. Taiwan also displayed effective epidemic prevention after reporting a seroprevalence of only 0.05% for the antibodies IgG and IgM, with no significant differences observed in sex, age, and the risk of seropositivity for SARS-CoV-2 [15]. In Brazil, a recently published cross-sectional study that included serological household surveys reported a pooled seroprevalence of 1.9% in the first measurement and 3.1% in the second measurement for IgG and IgM. In addition, no significant differences were found between men and women, with the highest seroprevalence observed in those aged between 20 and 59 years [18]. In a study to evaluate the seroprevalence among asymptomatic and paucisymptomatic participants, the overall SARS-CoV-2 among the sample was 8.83%, with significant differences observed in seropositivity rates between those who had contact history with a confirmed case compared to those who denied any close contact (59.9% vs 40.1%; p<0.001). Furthermore, hypertension, hypothyroidism, and asthma were the most associated comorbidities in the paucisymptomatic group (p= 0.05, p= 0.03, p= 0.006, respectively) [19]. In Gulf countries, a large population-based survey that included 17,457 Omani subjects reported that the seroprevalence ranged from 5.5% to 22% when conducted on 2 phases with nearly a 2-to 4-week gap in between. Gender was not a significant predeterminant of SARS-CoV-2, but significant differences were observed between different age groups. In addition, those who were in close proximity with contact people had a 96% higher risk of acquiring the disease compared to those who were not [20]. Another study conducted in the United Arab Emirates between July and August 2020 reported a seroprevalence of 10.4%. No differences were observed in gender, and national citizens had lower seroprevalences compared to foreign residents [21]. Locally, a study conducted this year that included 11,703 serum samples from six regions in Saudi Arabia revealed that the overall seroprevalence was 11%, with evident discrepancy across the regions. The highest seroprevalence was reported in Hail (20.8%) followed by Makkah (18.8%), and Jazan constituted the lowest seropositivity (1.5%) [22].

Due to the number of asymptomatic cases that go untested, the use of seroprevalence surveys has become of utmost importance, particularly to the national surveillance, to provide a more accurate measure of the overall prevalence of COVID-19 in the community and their current immune status. In addition, a low seroprevalence of SARS-CoV-2 specific antibodies reflects a well-implemented control measure. Thus, investigating the exact proportion of those who developed antibodies against the virus can help us identify immune gaps, evaluate the efficacy of current preventive measures, and direct public health decisions accordingly.

## Materials and methods

Aim

The aim of this study was to estimate SARS-CoV-2 seroprevalence among residents in seven regions in Saudi Arabia and to identify the epidemiological characteristics of discovered cases.

Study design

This is a cross-sectional study (community-based sero-survey).

Study area/setting

The study was conducted in seven regions in Saudi Arabia: Asir, Albaha, Najran, Jazan, Eastern Province, Makkah, and Madinah regions. All districts from the selected regions were included.

Data were collected in two phases: phase 1 covered the regions Asir, Albaha, Najran, and Jazan, and blood serums were collected during August 2020. Phase 2 covered the Eastern Province, Makkah, and Madinah regions, and blood serums were collected from late July till late October 2020.

Study subjects

Inclusion criteria included all individuals living in the same residential area (for the last six months) in the selected seven regions in Saudi Arabia, prior positive PCR result for COVID-19 and symptoms free for greater than or equal to 14 days, and never tested for COVID-19 and asymptomatic. Those previously labeled as positive COVID-19 cases (in less than 14 days) were excluded.

Sample size

Given that the seroprevalence estimates of SARS-CoV-2 ranged from 0.1% to 47%, we used an estimated proportion of 47% and precision of estimate 0.03 with 95% confidence interval [23]. Using the Epitools software to estimate a single proportion [24], our minimum sample size was estimated to be 1,064. In addition, a percentage of 20% was added to each area sample size to compensate for non-respondents. Moreover, additional 500 were added to the sample of regions with population over one million.

Sampling technique

According to data collection team, the sampling technique was multistage random consecutive sampling. From each region, random governates were selected, and all districts of selected governates were included. Mobile clinics were located near a mosque in each district, and people within the community were invited to participate until a sample proportionate to the population was reached. Proportionate sample was decided based on the total number of population registered in the primary health care center (PHCC) of each district (Table 1).

**Table 1:** Number of samples by region

Region/province	No. of population registered at a PHCC	No. of collected samples
Asir	13,889,030	2,544
Albaha	250,334	1,158
Jazan	1,068,240	1,486
Najran	517,070	1,158
Eastern province	2,628,041	2,890
Makkah	6,107,216	5,208
Medina	1,310,016	1,428
Total		15,872

Data collection methods, instruments used, and measurements

Blood samples were collected for serology and were tested at the Ministry of Health (MOH) laboratories. The data were collected by a survey team through mobile clinics managed by the MOH teams at the study site in the selected area (same place where the selected individuals live). The survey team consisted of survey coordinators, MOH staff trained for sample collection, assistants, and driver. The selected participants were then asked to complete an interviewer-administered questionnaire developed by the Public Health Authority (Weqaya) (see Appendix Table 8), which was piloted on a small sample for validity prior to approaching our participants. The questionnaire gathers information about participants' demographics (age, sex, etc.), notification information (details of area), clinical information (symptoms, time of onset, medical history, etc.), and epidemiological information (movements’ history, contact exposure, etc.). Afterward, 4 to 5 ml of blood sample was collected for serology and tested at MOH laboratories. The SARS-Cov-2 virus IgG/IgM antibody detection kits (ELISA) developed by BGI company (Shenzhen, China) was used by the MOH and was available at the mobile clinics for estimating the seroprevalence. The kit holds a general sensitivity of nearly 99% for IgG and IgM and a specificity of 96.76% for IgM and 98.38% for IgG [25]. Also, the positive predictive value was estimated to be 100 for both IgG and IgM antibodies, whereas the negative predicted value was 94.74% for IgG and 91.84% for IgM [26]. Seropositive cases were defined as those reporting a presence of reactive SARS-CoV-2 specific antibodies in their blood.

Statistical analysis

Data were entered and analyzed using SPSS version 25, and a p-value of <0.05 and 95% CI were used to report the significance. Also, descriptive statistical analysis was employed to describe the demographics of the study sample population. The numerical variable (age) was expressed as mean and standard deviation, and categorical variables were presented as frequency with proportions. Our main outcome (seroprevalence) is a categorical dichotomous outcome (reactive/non-reactive), which was expressed as the proportion of specimens that were confirmed reactive for SARS-CoV-2. Furthermore, the chi-square test was employed to test the association between the regions and the sample result and between the history of comorbidities and developing symptoms. In addition, binary logistic regression models were used to test the association between our main outcome variable and the clinical and epidemiological variables, and the magnitude of the association was presented as the odds ratio. Moreover, multivariate analysis was chosen as a method to adjust for suspected confounding factors.

## Results

Of the 17,000 intended samples, a total of 15,872 samples were retrieved and included in the analysis. The study subjects aged between 2 and 99 years were included in this analysis, with a mean age of 39.14 ± 13.998 years. More than half of the subjects were males (58.1%) vs. females (41.9%). Most of the participants were married (77.1%), and national citizens accounted for nearly 80% of the sample. The overall seroprevalence of SARS-CoV-2 in the sample was (17.0%) (Table 2).

**Table 2:** SARS-CoV-2 seroprevalence and socio-demographic characteristics of study participants ^1^1,754 (11.1) missing.

Variable		N (%)
Age	0-19	851 (5.4)
	20-34	5,726 (36.6)
	35-49	5,490 (35.1)
	50-64	2,747 (17.6)
	≥ 65	821 (5.3)
	Mean (SD)	39.14 ± 13.998
Sample result^1^	Non-reactive	11,422 (72.0)
	Reactive	2,696 (17.0)
Gender	Male	9,220 (58.1)
	Female	6,650 (41.9)
Marital status	Single	3,596 (22.9)
	Married	12,109 (77.1)
Nationality	Citizen	12,135 (78.2)
	Resident	3,380 (21.8)
Education	No education	2,119 (14.0)
	Primary	1,411 (9.3)
	Intermediate	1,636 (10.8)
	Secondary	3,714 (24.5)
	Diploma	1,418 (9.3)
	University and higher education	4,875 (32.1)
Region	Makkah	5,208 (32.8)
	Medinah	1,428 (9.0)
	Najran	1,158 (7.3)
	Asir	2,544 (16.0)
	Jazan	1,486 (9.4)
	Albaha	1,158 (7.3)
	Eastern province	2,890 (18.7)

Only around 9% of participants recalled having symptoms, of which headache was the most common (6%) followed by joint pain (5.3%) (Table 3).

**Table 3:** History and type of symptoms of study participants

Variable	Category	N (%)
Symptoms	No	14,486 (91.3)
	Yes	1,386 (8.7)
High temperature > 38°C	No	15,053 (95.1)
	Yes	774 (4.9)
Sore throat	No	15,017 (94.9)
	Yes	811 (5.1)
Runny nose	No	15,232 (96.2)
	Yes	595 (3.8)
Cough	No	15,095 (95.4)
	Yes	726 (4.6)
Shortness of breath	No	15,432 (97.5)
	Yes	398 (2.5)
Nausea or vomiting	No	15,534 (98.1)
	Yes	294 (1.9)
Loss of smell	No	15,329 (96.8)
	Yes	507 (3.2)
Headache	No	14,884 (94.0)
	Yes	943 (6.0)
Joint pain	No	14,988 (94.7)
	Yes	837 (5.3)
Muscle pain	No	15,085 (95.3)
	Yes	749 (4.7)
Diarrhea	No	15,484 (97.9)
	Yes	332 (2.1)

Regarding the history of contact, visiting hospitals or health facilities was the highest observed among this category; nearly 20% of participants visited such settings in the past 14 days, followed by going out and mixing with other people (9.6%). Only 8.1% reported being in contact with a confirmed or suspected case, and more than 5% reported attending public gatherings in the past two weeks (Table 4).

**Table 4:** Contact history of study participants

Variable	Category	N (%)
During the 14 past days, did the participant have contact with a confirmed or suspected case of COVID-19 infection or a condition showing symptoms of a respiratory infection?	No	13,990 (91.9)
	Yes	1,227 (8.1)
During the past 14 days, did the participant go out of the house and mix with people a lot?	No	13,937 (90.4)
	Yes	1,486 (9.6)
During the past 14 days, did the participant visit any health facility (clinic, hospital, hospitalization, waiting rooms in medical facilities)?	No	12,430 (80.5)
	Yes	3,013 (19.5)

Furthermore, the chi-square table (Table 5) shows a significant association between the regions and sample result (p<0.001). Makkah region constituted the highest prevalence of reactive cases (33.3%) followed by Asir (19%). The lowest seroprevalence of SARS-CoV-2 was detected in Albaha, with only (1.3%) reactive cases.

**Table 5:** Seroprevalence of SARS-CoV-2 by region

Variable		Non-reactive	Reactive	Chi-square	P-value
		N (%)	N (%)		
Region	Makkah	4,021 (35.2)	899 (33.3)	520.053	<0.001
	Medinah	1,148 (10.1)	280 (10.4)		
	Najran	940 (8.2)	133 (4.9)		
	Asir	1,685 (14.8)	518 (19.2)		
	Jazan	1,161 (10.2)	130 (4.8)		
	Albaha	968 (8.5)	35 (1.3)		
	Eastern province	1,499 (13.1)	701 (26)		
Total		11,422	2,696		

Regarding the history of comorbidities, diabetes (9.6%) and hypertension (7.9%) were the most prevalent diseases in the sample. Immunodeficiency disorders were the least observed among the participants (0.3%). In addition, only a small proportion of participants reported having symptoms (8.7%), and there was a significant association between all comorbidities and developing symptoms except for diabetes (p=0.668) (Table 6).

**Table 6:** Crosslinking between the history of comorbidities and having symptoms *Percentages are presented as within the outcome.

Variable	Category	Without symptoms	With symptoms	Chi-square	P-value
		N (%)*	N (%)		
Heart disease	No	14,259 (98.9)	1,353 (97.7)	15.183	<0.001
	Yes	161 (1.1)	32 (2.3)		
Total		14,419	1,385		
Hypertension	No	13,315 (92.3)	1,250 (90.3)	7.388	0.007
	Yes	1,109 (7.7)	135 (9.7)		
Total		14,424	1,385		
Diabetes	No	13,030 (90.4)	1,247 (90)	0.184	0.668
	Yes	1,385 (9.6)	138 (10)		
Total		14,415	1,385		
Chronic kidney disease	No	14,287 (99.2)	1,358 (98.1)	17.741	<0.001
	Yes	118 (0.8)	27 (1.9)		
Total		14,405	1,385		
Chronic liver disease	No	14,378 (99.7)	1,374 (99.3)	10.491	0.001
	Yes	40 (0.3)	11 (0.8)		
Total		14,418	1,385		
Immunodeficiency disorder	No	14,382 (99.8)	1,368 (98.8)	37.692	<0.001
	Yes	31 (0.2)	16 (1.2)		
Total		14,413	1,384		
Asthma	No	14,388 (99.4)	1,370 (98.8)	7.113	0.008
	Yes	82 (0.6)	16 (1.2)		
Total		14,470	1,386		

Using the enter method in univariate logistic regression, Table 7 showed that all covariates were significantly associated with the sample result, except mixing with people in the past 14 days and testing reactive for SARS-CoV-2 (p=0.603). Those aged 20-34, 35-49, and 50-64 years had a significantly higher risk of being seropositive when compared to those aged 0-19 years (p<0.05). Also, females were 18.4% less likely to test reactive for SARS-CoV-2 compared to males, this finding was significant at p<0.001. Furthermore, nationality, educational level, region, and the month of sample collection were all significantly associated with seropositivity in the univariate model (p<0.001). Regarding the history of contact, subjects who had contact with a confirmed or suspected case of COVID-19 and those who visited hospitals and health facilities in the past 14 days were also significant factors in the sample result. In the multivariate model, after the adjustment of significant covariates, age was still a predeterminant for testing reactive for SARS-CoV-2 with the highest risk observed in the age group of 20-34 years, being nearly 1.5 times more likely to test reactive compared to the reference group (OR= 1.482; 95% CI: 1.163-1.889; p=0.001), followed by those aged 35-49 years (OR=1.320; 95% CI: 1.035-1.684; p=0.025). No significant association was found between older adults aged 50-64 years and the elderly group aged ≥ 65 years (p>0.05). Also, gender and visiting health facilities in the last 14 days were no longer significant after adjustment (p=0.273 and p=0.248). Regarding education, those with lower education levels were at more risk of being seropositive compared to those with university and higher levels, with the highest risk being across primary level and (OR= 1.712; 95% CI: 1.311-1.813; p<0.001) and intermediate level (OR: 1.702; 95% CI: 1.446-2.003; p<0.001). Furthermore, residents were over two times at more risk of being reactive to SARS-CoV-2 compared to citizens (OR=2.283; 95% CI: 2.041-2.554; p<0.001). Regarding region, the Eastern province had the highest risk when compared to the Makkah region (OR=2.275; 95% CI: 1.976- 2.619; p<0.001). Last, in the multivariate model, subjects who were in contact with a confirmed or suspected case increased their odds of being seropositive by nearly 1.5 times (OR= 1.499; 95% CI: 1.264-1.778; p<0.001).

**Table 7:** Seropositivity of SARS-CoV-2 by general demographics and contact history

Variable	Category	Univariate analysis			Multivariate analysis		
		OR	95% CI	P-value	aOR	95% CI	P-value
Age (reference: 0-19)							
	20-34	1.566	1.259- 1.948		1.482	1.163- 1.889	0.001
	35-49	1.485	1.193- 1.849		1.320	1.035- 1.684	0.025
	50-64	1.396	1.108- 1.758		1.164	.901- 1.504	0.244
	≥65	1.091	0.817- 1.456		1.091	0.792- 1.502	0.593
Gender (reference male)	Female	0.816	0.749- 0.890		0.946	0.857- 1.045	0.273
Education level (reference university and higher)							
	No education	1.310	1.140- 1.506		1.542	1.311- 1.813	
	Primary	1.577	1.351- 1.841		1.712	1.442- 2.033	
	Intermediate	1.732	1.500- 2.000		1.702	1.446- 2.003	
	Secondary	1.254	1.113- 1.413		1.339	1.175- 1.526	
	Diploma	1.204	1.021- 1.420		1.320	1.090- 1.568	0.003
Nationality (reference citizen)	Resident	2.194	1.998- 2.409		2.283	2.041- 2.554	
Region (reference Makkah)							
	Medinah	1.091	0.940- 1.267		0.900	0.752- 1.077	0.250
	Najran	0.633	0.520- 0.769		0.578	0.452- 0.740	
	Asir	1.375	1.217- 1.554		1.149	0.955- 1.383	0.141
	Jazan	0.501	0.412- 0.609		0.581	0.454- 0.745	
	Albaha	0.162	0.115- 0.228		0.147	0.100- 0.214	
	Eastern province	2.049	1.774- 2.367		2.275	1.101- 1.583	
Month of sample collection (reference July 2020)							
	August 2020	0.342	0.136- 0.860		0.342	0.134- 0.872	0.025
	September 2020	0.489	0.192- 1.244		0.342	0.133- 0.880	0.026
	October 2020	0.552	0.220- 1.385		0.367	0.145- 0.931	0.035
During the 14 past days, did the participant have contact with a confirmed or suspected case of COVID-19 infection or a condition showing symptoms of a respiratory infection? (reference No)	Yes	1.569	1.358- 1.813		1.499	1.264- 1.778	
During the past 14 days, did the participant visit any health facility (clinic, hospital, hospitalization, waiting rooms in medical facilities)? (reference No)	Yes	0.877	0.786- 0.978	0.019	0.927	0.815- 1.054	0.248

## Discussion

This study explored the seroprevalence and identified the epidemiological characteristics of SARS-CoV-2 in Saudi Arabia. The findings of this study revealed that the seroprevalence was 17%, with large discrepancies between the regions. Also, age, educational level, nationality, having symptoms, and region of residence were significant predeterminants of seropositivity. Furthermore, being in contact with a confirmed or suspected case significantly increased the risk of testing reactive for SARS-CoV-2.

In this study, serum samples were extracted from 15,872 participants from different regions across the kingdom. The results of this study found the overall seroprevalence of SARS-CoV-2 in Saudi Arabia to be 17%, ranging from 1.3% to 33% across different regions. This proportion is notably higher than what has been reported in the U.S. and Eastern countries [11,13,15,17]. It is worth mentioning that most of these studies were conducted in early 2020 before most countries were affected by the pandemic. Hence, the true proportion might be underestimated due to the small number of people infected at that time. In addition, when compared to gulf countries that conducted their serological surveys around the same time as this study, both the Emirati and the Omani study reported lower seroprevalences. However, during the second phase of which they repeated the serologic survey at a later time, Oman showed a higher proportion compared to our finding. Thus, given that time of data collection largely affects the seroprevalence, it might not be appropriate to compare countries with different incidence rates.

Several studies suggested that asymptomatic patients have lower levels of antibodies and might not become seropositive until two months post-infection [27]. Hence, asymptomatic participants who got their serum samples recently collected from the time of infection might go undetected and consequently underestimating the true seroprevalence. Furthermore, the incidence o SARS-CoV-2 in Saudi Arabia was higher in August and decreased around the end of October [28]. However, we observed lower seropositivity in the regions that got their samples collected during the month of August (phase I) compared to those collected in later months (phase II). This finding could further suggest that recent incident cases had lower antibody levels and thus were not recognized under the seroprevalence survey. Also, the seroprevalence in this sample is notably higher than what has been detected in the previously published Saudi study [22]. This could be due to the different regions included in the sample with different incidence rates. Furthermore, seroprevalence surveys are largely influenced by the timing of sample collection. Given that the incidence of SARS-CoV-2 in Saudi Arabia peaked around late June 2020 [28], and the data collection for that study was also during June for most regions, most asymptomatic COVID-19 cases reported at that time would go undiscovered as they have not developed sufficient level of antibodies yet and hence may underestimate the true proportion.

Regarding history of comorbidities, similar association was found in previous studies with respect to asthma and hypertension and developing symptoms [19,22]. Diabetes mellitus was the only comorbidity in our sample that was not significantly associated with having symptoms. This result is contrary to what has been found in some studies, suggesting that diabetes is one of the main predeterminants of experiencing COVID-19 symptoms and having severe disease progression [29,30]. This finding could be influenced by the population’s general awareness of the complications and severity of COVID-19 in those with diabetes. Consequently, diabetic individuals might refrain more from going out and thus have lower probability of contracting the virus. Also, regarding having symptoms or not, the variable incorporated in testing this association might not be particular to COVID-19 symptoms only. Hence, the actual proportion of cases experiencing symptoms might be overestimated. Similar to previous findings [13,15-17,31], we did not find any differences between males and females in our sample. Furthermore, regarding age, our finding was aligned with what has been demonstrated in previous studies, that is younger adults and adults (20-34 and 35-49 age group) accounted for the highest seropositivity when compared to the youngest age group (0-19 years) [16,18]. Also, citizens were found to have lower seroprevalence compared to residents. These findings are in accordance with findings reported by the Emirati study [21], which suggests that national citizens might be more attentive to preventive precautions compared to residents.

Last, with respect to subjects’ history of contact, similar to previous reports, close contact with a case was found to be an independent risk factor for being seropositive [20,32], However, we were not able to replicate in our study the previously reported association that visiting a hospital or a health facility increased the risk of testing reactive for SARS-CoV-2 antibodies [13]. This could be attributed to Saudi Arabia’s strict preventive measures in health settings.

Strengths and limitations

To our knowledge, this is the largest multi-regional community-based study of SARS-CoV-2 seroprevalence in Saudi Arabia. Furthermore, the results go beyond previous reports showing a cross-linking between seropositivity and various risk factors. In addition, this study demonstrates the feasibility of conducting a serological survey within the community via mobile clinics. Also, the use of highly sensitive and specific sero-essay test adds to the strengths of this study. However, this study has its limitation. First, since participants are required to remember their contact exposure during the past 14 days, the results might be subjected to recall bias. Also, given that serum samples were collected at different months for different regions, it might not be appropriate to compare the seroprevalence between regions since incidence rates have dropped in later months. Another limitation is that not all regions were included in this study, which further threatens our external validity. In addition, this study did not gather information on some major confounding factors such as occupation, lifestyle habits, and travel history; hence, the inability to adjust for these factors might expose our results to cofounding bias.

Repeated seroprevalence surveys are essential to track the current status of the epidemic and monitor the immunity of the population. With the introduction of vaccines and mandatory COVID-19 vaccination strategy in the kingdom, we recommend that the estimates provided in this study be updated by future studies to better reflect the current immune status of the population. Furthermore, given that the findings of this study indicate that the transmission of SARS-CoV-2 is dependent on close contact, social distancing and preventive measures must be emphasized particularly as we are still in the midst of the pandemic.

## Conclusions

In conclusion, this study estimates the national seroprevalence of SARS-CoV-2 in Saudi Arabia to be 17%, with large discrepancies between the regions. At the time of this study, most of the population did not have the SARS-CoV-2 specific antibodies. This suggests that the population is still below the threshold of herd immunity and hence emphasizes the importance of mass vaccination programs and abiding by recommended prevention precautions (wearing masks, social distancing, and self-quarantine for cases and their contacts). Such measures are crucial to controlling the further impact of the pandemic till the population reaches herd immunity.

## Appendices

Verbal Consent

ورقة معلومات للمشارك في المسح العشوائي

فحص مرض فيروس كورونا المستجد (كوفيد 19)

فيروس (كورونا المستجد) من فصيلة فيروسات كورونا؛ حيث ظهرت أغلب حالات الإصابة به في مدينة ووهان الصينية نهاية ديسمبر ٢٠١٩م على صورة التهاب رئوي حاد. هو فيروس جديد يشكل تحديا كبيرا للصحة العامة في مختلف دول العالم. وقد نفذت دول مختلفة سياسات التباعد الاجتماعي و تقييد الحركة في المقاطعات والمدن أو حتى على الصعيد الوطني. وقد تم تنفيذ هذه الإجراءات دون خطة لكيفية تخفيف هذه القيود. ولهذا المعلومات التي سيقدمها هذا المسح سيكون لها أثر كبير في وضع الخطط المناسبة لتخفيف القيود في وطننا الغالي.

في هذا المسح , سيتم جمع عينة دم الوريد ليتم فحصها في المختبرات والتأكد من خلوها من أو وجود أجسام مضادة لفايروس كورونا المستجد في الدم. كذلك سيتم تعبئة استبيان حول عوامل التعرض لمرض فيروس كورونا الجديد.

هذا المسح تحت اشراف وزارة الصحة والاشتراك فيه اختياري ولن يؤثر قرارك بعدم الاشتراك على العناية الطبية المستحقة. وسيتم ابلاغكم لاحقاً بنتيجة العينة, وفي حال ثبتت ايجابية العينة سيتم التواصل معكم من قبل فرق الصحة العامة .

نسأل الله لكم تمام الصحة والعافية ونشكر تعاونكم. واذا كان لديكم اي اسئلة تتعلق بهذا المسح فبإمكانكم الاتصال على  937

English Translation

Information sheet for the participant

Examination for the emerging Corona Virus Disease (COVID-19)

The (emerging corona) virus is from the family of coronaviruses; Most of its cases appeared in the Chinese city of Wuhan at the end of December 2019 in the form of acute pneumonia. It is a new virus that poses a major challenge to public health in various countries of the world. Many countries have implemented social distancing and movement restriction policies in provinces, cities or even nationwide. These measures have been implemented without a plan for how to ease these restrictions. Therefore, the information that this survey will provide will have a great impact on developing appropriate plans to ease restrictions in our dear homeland.

In this survey, a blood sample will be collected and examined in the laboratories to assess the presence of antibodies to the emerging corona virus in the blood. A questionnaire will also be filled out regarding factors of exposure to the novel coronavirus disease.

This survey is under the supervision of the Ministry of Health and participation in it is completely optional. Your decision not to participate will not affect the medical care you are entitled to. You will be notified later with the sample result, and if the sample proves positive, the public health teams will contact you.

We ask God to grant you complete health and wellness, and we thank you for your cooperation. If you have any questions related to this survey, you can call 937.

**Table 8:** COVID-19 Community Survey Questionnaire

Patient Information: Patient					
Name:		Date of birth:			
Marital status:		Age:			
National classification:	( ) Citizen ( ) Resident	Gender:		( ) Male ( ) Female	
Official evidence number$:		Mobile number:			
Address	House Number ( ), Street Name ( ), Neighborhood ( ) City ( ) Region/Governorate ( )				
Education					
Clinical information: the first date of symptoms (if any)	(Gregorian day/month/year): / /				
Symptoms	Yes	No	Symptoms	Yes	No
High temperature ( >38°C)			Nausea and/or vomiting		
History of high temperature (unmeasured)			Loss of smell		
Sore throat			Headache		
Runny nose			Joint pain		
Cough			Muscle pain		
Shortness of breath			Diarrhea		
As well mention any other symptoms:					
